# The Output Signal of Purkinje Cells of the Cerebellum and Circadian Rhythmicity

**DOI:** 10.1371/journal.pone.0058457

**Published:** 2013-03-07

**Authors:** Jérôme Mordel, Diana Karnas, Paul Pévet, Philippe Isope, Etienne Challet, Hilmar Meissl

**Affiliations:** 1 Neuroanatomical Department, Max Planck Institute for Brain Research, Frankfurt/M, Germany; 2 CNRS UPR3212, Institute for Cellular and Integrative Neuroscience, Strasbourg, France; Tokyo Medical and Dental University, Japan

## Abstract

Measurement of clock gene expression has recently provided evidence that the cerebellum, like the master clock in the SCN, contains a circadian oscillator. The cerebellar oscillator is involved in anticipation of mealtime and possibly resides in Purkinje cells. However, the rhythmic gene expression is likely transduced into a circadian cerebellar output signal to exert an effective control of neuronal brain circuits that are responsible for feeding behavior. Using electrophysiological recordings from acute and organotypic cerebellar slices, we tested the hypothesis whether Purkinje cells transmit a circadian modulated signal to their targets in the brain. Extracellular recordings from brain slices revealed the typical discharge pattern previously described *in vivo* in single cell recordings showing basically a tonic or a trimodal-like firing pattern. However, in acute sagittal cerebellar slices the average spike rate of randomly selected Purkinje cells did not exhibit significant circadian variations, irrespective of their specific firing pattern. Also, frequency and amplitude of spontaneous inhibitory postsynaptic currents and the amplitude of GABA- and glutamate-evoked currents did not vary with circadian time. Long-term recordings using multielectrode arrays (MEA) allowed to monitor neuronal activity at multiple sites in organotypic cerebellar slices for several days to weeks. With this recording technique we observed oscillations of the firing rate of cerebellar neurons, presumably of Purkinje cells, with a period of about 24 hours which were stable for periods up to three days. The daily renewal of culture medium could induce circadian oscillations of the firing rate of Purkinje cells, a feature that is compatible with the behavior of slave oscillators. However, from the present results it appears that the circadian expression of cerebellar clock genes exerts only a weak influence on the electrical output of cerebellar neurons.

## Introduction

Anticipation of daily and seasonal environmental rhythms is provided by a biological clock that controls the circadian rhythm of physiological, endocrine and behavioral processes. The dominant pacemaker is located in the hypothalamic suprachiasmatic nucleus (SCN) and is composed of numerous individual clock cells which are synchronized to solar time by direct retinal afferents [1]. However, rhythmically expressed clock genes which are responsible for the sustained 24 hour oscillations in the SCN were also discovered in other brain areas and in many peripheral tissues [2], [3]. It is believed that the mammalian circadian timing system is composed of a hierarchical organized network of oscillators involving the entrained master oscillator in the SCN and a number of slave oscillators in other brain areas and in peripheral organs [1]. Circadian gene expression in peripheral tissues, which are themselves not light sensitive and can be entrained by nonphotic cues, depend to a large extent on a functional SCN pacemaker in intact animals [4]. Whereas the light-dark cycle is the most important zeitgeber for the master clock in the SCN, time of feeding is the dominant zeitgeber for peripheral tissues.

The food entrainable oscillator (FEO) is responsible for the food anticipatory activity (FAA) that precedes the mealtime during scheduled feeding in mammals [5], [6]. The localization of the presumptive FEO was assessed by lesioning specific brain areas and measuring the reduction of the FAA. From these studies it was assumed that the FEO may consist of a network of coupled brain regions involving principally hypothalamic areas outside of the SCN, including the dorsomedial hypothalamus, and also the brainstem with the parabrachial nucleus [7], [8], [9], [10]. Interestingly, restricted feeding induces phase-shifts of rhythmic clock gene expression in both regions without affecting expression of the same clock genes in the SCN [9]. Circadian rhythms in the SCN are only affected when the timed feeding becomes additionally hypocaloric [11]. This suggests that the FEO is independent from the SCN, and possesses a self-sustained clock mechanism.

Another possible candidate involved in a feeding entrained network is the cerebellum which shows, besides its established control of fine locomotor activity [12], a rhythmic expression of clock genes [13]. Destruction of Purkinje cell function by an immunotoxin leads, similar as in mouse mutants with impaired cerebellar circuitry, to a strong diminution of rhythmic FAA which shows that the cerebellum belongs to a network of self-sustained FEO [13]. Rhythmic clock gene expression in the cerebellum is independent from the master clock in the SCN, because in cerebellar brain slices that are isolated from any input signal this rhythmicity persists for several days [3], [13]. However, if Purkinje cells harbor an intrinsic circadian oscillator, it is uncertain whether this rhythmic clock gene expression is transduced into a rhythmic neuronal output signal that can influence other brain targets involved in feeding behavior.

In the SCN, the circadian expression of clock genes forms the core of circadian rhythm generation and this intrinsic timekeeping signal must be transmitted to the SCN targets in the brain in the form of humoral or neural outputs [14]. Rhythmic SCN electrical activity as a circadian output signal can be recorded *in vivo* [15], as well as *in vitro* in dissociated cell cultures [16], [17], acute slices [18] or organotypic slice cultures [19], [20]. The activity of SCN output neurons is thus a reliable signal that communicates temporal information to various brain regions. In the present work, we investigated whether the circadian expression of clock genes in the cerebellum is also communicated as a rhythmic electrical output signal to brain areas. Since Purkinje cells provide the sole output signal of the cerebellar cortex we performed random single cell extracellular recordings in acute slices, as well as long-term MEA recordings from organotypic slices to elucidate whether the circadian oscillation of genes in the cerebellar clock is transduced into a circadian electrical output signal. Additionally, we investigated, using whole-cell patch-clamp recordings, a possible circadian modulation of synaptic inputs to Purkinje cells. Cerebellar Purkinje cells are firing spontaneously with high frequencies in the range of 20 to 50 Hz *in vivo* [21]. Spontaneous activity can be recorded with MEAs in acute slices [22] and also in organotypic slice cultures [23]. However, despite the considerable work already done on Purkinje cell physiology, we do not know whether the recently detected circadian clock gene expression is communicated as rhythmic electrical information to the brain.

## Materials and Methods

### Animals and Ethics Statement

Acute brain slices and organotypic slices were prepared from wild type mice (C57Bl/6). Animals were housed under a 12∶12 light/dark (LD) cycle with lights on at 7 am and lights off at 7 pm. All animal procedures were carried out in accordance with institutional guidelines of the Max Planck Institute for Brain Research, Frankfurt, and the University of Strasbourg, following the standards described by the German animal protection law (Tierschutzgesetz), the rules of the European Committee Council Directive of November 24, 1986 (86/609/EEC) and the French Department of Agriculture (licence no. 67-7 and 67–88). Killing of mice for organ harvesting (brain slices) has been approved by the animal welfare officer of the respective facility (Max Planck Institute for Brain Research, Frankfurt) and reported to the local authorities (Regierungspraesidium Darmstadt).

### Preparation of Brain Slices

#### Acute brain slices

Animals (3–5 week old) were deeply anesthetized with isoflurane (CuraMed Pharma, Karlsruhe, Germany), the cerebellum was dissected out and placed in cold artificial cerebrospinal fluid (aCSF) (4°C) bubbled with carbogen (95% O2, 5% CO_2_), containing in mM: NaCl 120; KCl 3; NaHCO_3_ 26; NaH_2_PO_4_ 1.25; CaCl_2_ 2.5; MgCl_2_ 2; glucose 10; minocyclin 0.00005 (Sigma-Aldrich). Transverse slices of 330 µm thickness were prepared with a vibrating blade microtom (Microm HM 650V, Thermo Scíentific) in potassium-based medium, containing in mM: K-gluconate 130; KCl 14.6; EGTA 2; HEPES 20; glucose 25; minocyclin 0.00005; D-(-)-2-amino-5-phosphonovaleric acid (D-AP5) 0.05 [24]. After cutting, slices were soaked a few seconds in a sucrose-based medium at 34°C, containing in mM: Sucrose 230; KCl 2.5; NaHCO_3_ 26; NaH_2_PO_4_ 1.25; glucose 25; CaCl_2_ 0.8; MgCl_2_ 8; minocyclin 0.00005; D-AP5 0.05. Slices were maintained in bubbled aCSF.

#### Organotypic slices

Mouse pups of age P0 to P3 were rapidly decapitated, and the brain was removed and placed in ice-cold aCSF complemented with 100 µg/ml penicillin/streptomycin. Coronal and sagittal slices of 250–350 µm were cut on a vibratome, and placed on a Millipore culture insert (MilliCell-CM) in a 35-mm culture dish with a small amount of culture medium (ca. 1 ml). The medium consisted of DMEM/F12 (Invitrogen, Karlsruhe, Germany) supplemented with 10% fetal calf serum, 2.5 mM glutamax (Invitrogen, Karlsruhe), 10 mM HEPES (Sigma-Aldrich, München, Germany) and 100 µg/ml penicillin/streptomycin (Invitrogen, Karlsruhe). Medium was exchanged three times per week. The dishes were incubated at 37°C in 5% CO_2/_95% air for at least two weeks. Before recording, the membrane of the culture insert was cut to approximately the size of the slice. The slice was then inverted with the culture membrane at the top and placed onto a nitrocellulose coated MEA (Multi Channel Systems, Reutlingen, Germany). Organotypic slices were maintained on MEAs for 1–3 weeks under continuous superfusion with recording medium.

### MEA Recordings

Long-term recordings of the firing rate from organotypic brain slices were carried out with a MEA-1060 recording system (Multi Channel Systems, Reutlingen) as previously described [25]. Two types of high density MEAs (HD-MEA) and one standard type with different electrode layouts were used. One HD-MEA type consisted of 2 fields with 30 electrodes each with a diameter of 10 µm and 30 µm spacing. The distance between the two fields was 500 µm. The second HD-MEA type consisted of one field of 60 electrodes with 10 µm diameter and 40 µm spacing between the electrodes. It was possible to record the activity of the same Purkinje cell on several electrodes of the HD-MEAs. The third standard type consists of 60 electrodes of 30 µm diameter evenly spaced by 200 µm. This type of MEA allowed to record simultaneously from different cells located at various places in the cerebellar cortex.

Extracellular voltage signals recorded from the MEA electrodes were amplified ×1200 and sampled at 32 kHz on 60 channels simultaneously. Extracellularly recorded spikes which are usually embedded in biological and thermal noise of about 15 µV peak to peak were detected by using a threshold based algorithm. Action potentials exceeding a defined voltage threshold were digitized and stored as time-stamped spike cut-outs using the MC_Rack software (Multi Channel Systems).

Recording medium consisted of DMEM/F12 with the same supplements as the culture medium with the exception that the HEPES content was elevated to 20 mM and the NaHCO_3_ levels reduced to 0.56 g/l. During long-term recordings, medium was exchanged continuously at a flow rate of 20 µl/min using a SP 260PZ syringe pump (WPI, Sarasota, USA). Culture chamber, application system and inflow-, outflow-system were completely sealed to prevent bacterial contamination.

### Patch-clamp Recordings

Extracellular spike recordings were made with borosilicate pipettes (Warner Instruments, USA) filled with 3 M NaCl and 20–30 MΩ resistance using a multiclamp 700A amplifier and WinWCP 4.2.x freeware (John Dempster, SIPBS, University of Strathclyde, UK). Purkinje cells were selected for recording under visual guidance of differential interference contrast microscopy (BX51, Olympus). Recordings were performed at room temperature from randomly selected Purkinje cells with a sampling rate of 20 kHz. The activity of each randomly selected cell was recorded for 5 minutes. Recordings from each slice were conducted for a maximum time of 1 h, before the next slice was used. For the same preparation a maximum of 4 slices were used which should limit the risk that a degradation of the health of the slices could influence the firing rate. The electrical activity of the cells was recorded independently of their position in the cerebellar folia. All measurements were completed within 4 hours following decapitation.

Whole-cell recordings were performed at room temperature with an EPC-9 patch-clamp amplifier and Pulse 8.11 software (HEKA Elektronik, Lamprecht, Germany). The patch pipettes were pulled from borosilicate glass tubing (Hilgenberg, Malsfeld, Germany) on a horizontal puller and had a resistance of 5–8 MΩ. The pipette solution contained in mM: Cs-gluconate 125, CaCl_2_ 1, EGTA 10, MgSO_4_ 4.6, Na-HEPES 10, Na-ATP 4, Na-GTP 0.4, QX-3145 5 (pH 7.3, adjusted with CsOH). Purkinje neurons were identified by differential interference contrast microscopy (Axioscope 2, Zeiss). Input resistances typically were between 600 and 1400 MΩ. Series resistances were 10–20 MΩ and left uncompensated. The cell and pipette capacitances were cancelled. The liquid junction potential of the aCSF with regard to the pipette solution was approximately 15 mV. The holding potential was corrected for the junction potential. The signals were filtered at 1 kHz with an eight-pole Bessel filter built into the EPC-9 amplifier and digitized. The sampling rate was 10 kHz. Spontaneous inhibitory post-synaptic currents (IPSCs) were measured using Mini Analysis 6.0.3 software (Synaptosoft, Decatur, GA, USA).

### Immunohistochemistry

After MEA recordings, organotypic slices were gently removed from the electrode fields and indirect immunohistochemistry was performed on the free-floating slices. Slices were fixed for 10 min with 4% paraformaldehyde in 0.1 M phosphate buffer (PB) at room temperature, washed three times with phosphate buffer and then incubated overnight with a rabbit primary antibody, raised against either calbindin D28K (Cat. No. CB 38; Swant, Marly, Switzerland) to label Purkinje cells or calretinin (Cat.No. 7699/3H; Swant, Marly, Switzerland) to label granule cells. Primary antibodies were diluted to 1/200 in 0.1 M PB containing 10% normal goat serum, 1% bovine serum albumin, 0.5% Triton X-100, and 0.05% NaN_3_. After incubation overnight at room temperature, slices were washed out three times in PB 0.1 M. Then, slices were incubated for 1.5 hours at room temperature with the secondary, anti-rabbit antibody raised in goat, coupled with Alexa 488 and diluted to 1/500 in the same solution as the first antibody. Additional incubation for five minutes with DAPI (1/1000 in 0.1 M PB) was realized after wash out of the secondary antibody. Slices were mounted on microscope slides and coverslipped with Aqua Polymount medium (Polysciences, Warrington, PA). The labeled cerebellum neurons were examined with an Axioplan 2 microscope (Zeiss).

### Drug Application

Drugs were applied by superfusion through a multibarrel, pressure driven system (DAD12, ALA Scientific Instruments). The tip of the micromanifold had an inner diameter of 200 µm and was placed within 100 µm of the soma of the recorded cell. GABA and glutamate were applied for five seconds at different concentrations. The sequence of drugs applied to cells was randomized. The slices were continuously superfused at the same rate before and after drug application. Between drug applications, the cells were perfused for at least 3 min with aCSF to prevent possible receptor desensitization.

### Data Analysis and Statistics

All normally distributed data are presented as mean ± standard error of the mean. One-way analyses of variance (ANOVA) for repeated and independent measures were used to compare the firing rates and currents recorded at different Zeitgeber times in acute slices. Significant periodicity of neuronal activity measured on MEAs was determined with Fisher periodograms (Sigmastat 3; Systat Software). Statistical analysis of circadian phase and amplitude of electrical activity was performed by fitting a single cosinor model onto the data points (Time Series Analysis Seriel Cosinor: Expert Soft Technology, Esvres, France), by comparing the peak time of the firing rhythm and by using the ½ maximal rise-time of the activity rhythm as reference point. All different methods to quantify the phase shifts gave consistent results.

### Solutions and Chemicals

All drugs were purchased from Sigma-Aldrich (Taufkirchen, München, Germany) unless otherwise stated.

## Results

### Extracellular Recordings from Acute Cerebellar Slices

In order to assess a possible circadian component in the output signal of Purkinje cells, as it is inferred by the rhythmic expression of clock genes, extracellular single unit recordings were performed in sagittal cerebellar slices. For these long-term experiments, spanning a time-period of more than 24 h, we adopted the classical protocol used by Green and Gillette [26] to monitor circadian activity in the SCN under *in vitro* conditions. This protocol is based upon rapid and random sampling of single units for periods of 5 minutes, rather than the long-term activity of a single cell, to obtain the firing pattern of the ensemble of SCN neurons [18]. Using the same method, we sampled the mean firing rate of all Purkinje cells recorded, averaged the data and plotted them over the course of the 24 hour cycle.

**Figure 1:**
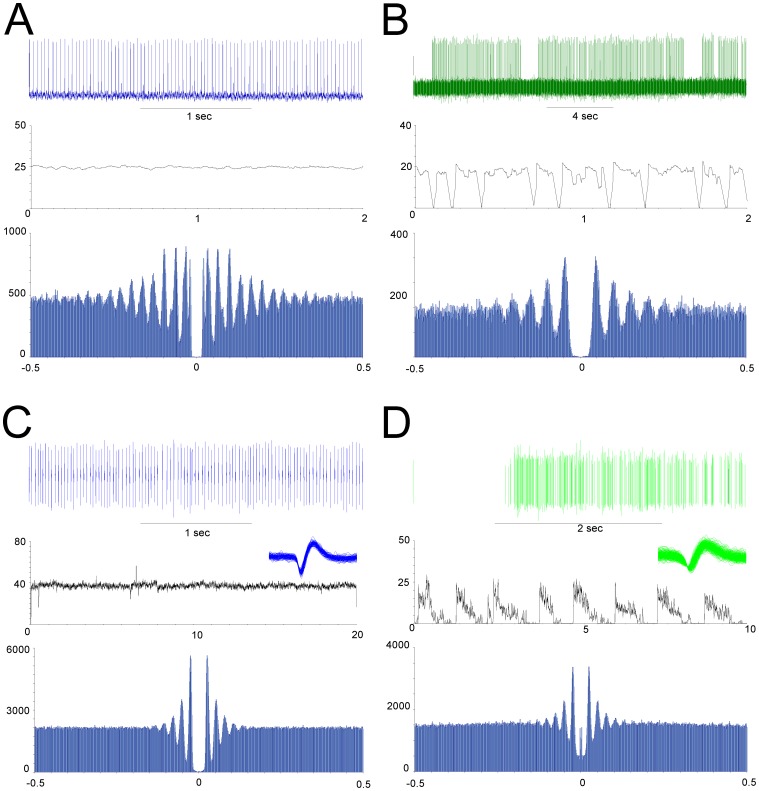
Firing pattern of Purkinje cells in single cell extracellular recordings (A and B) or MEA recordings (C and D). The main firing pattern observed were a tonic pattern (**A** and **C**, in blue) or a trimodal-like pattern consisting of a period of higher activity followed by a phase of tonic activity and a period of silence (**B** and **D**). Purkinje cell activity was comparable with both recording methods. For each example, a short portion of spike recordings (top trace), the firing rate (middle trace) and the auto-correlogram of the single neuron (bottom trace) are represented. The peaks in the correlograms show that cells are spiking with a regular period. Insets in (**C**) and (**D**) depict the waveform of averaged single units.

**Figure 2:**
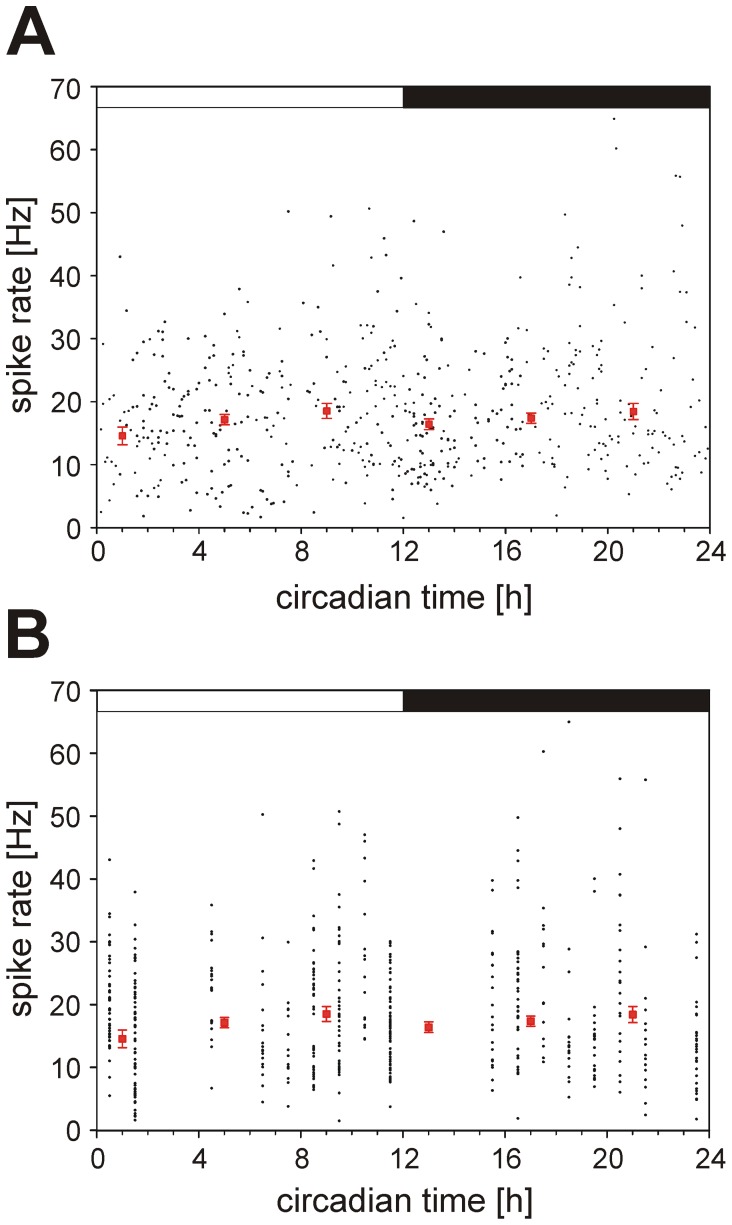
Extracellular recording from Purkinje cells in acute slices. **A)** Firing rate of 524 individual Purkinje cells were measured at different time of the day within four hours following decapitation. **B)** The same cells as in (**A**) were clustered corresponding to the time of decapitation. No rhythm can be observed between the different circadian times (p>0.05). Black dots represent the firing rates of every single Purkinje cell randomly recorded in the course of a day. Red squares with error bars show the median firing rate of all cells recorded in four hours. The black and white bars at the top of each group represent the periods of subjective day and night.

Extracellular recordings from visually identified Purkinje cells in acute slices showed similar firing rates as it is known for simple spikes from *in vivo* recordings [27]. The most frequent cell type displayed a trimodal-like pattern consisting of three successive phases with a phase of inactivity, a phase with bursting activity and a phase with a regular, tonic firing (Fig. 1B). The second type showed tonic and constant firing (Fig. 1A) and a third group of cells exhibited firing with random patterns (data not shown). The average firing rate of all 524 Purkinje cells recorded in this study was 18.64±0.43 Hz (min: 1.52 Hz; max: 64.89 Hz), which corresponds to the spike rate that is usually recorded in acute slices at room temperature. However, the mean firing rate of Purkinje cells did not show any clear circadian variation during the 24 hour cycle (p>0.1; Fig. 2A). When only cells with a similar firing pattern were taken into account, i.e. only cells with trimodal-like pattern (firing rate: 14.0±0.4 Hz) or only cells with tonic pattern (firing rate: 17.1±0.8 Hz), no significant circadian changes of the firing rate were observed (data not shown). Furthermore, in cells with trimodal-like pattern, the average duration of the silent phases (in the mean 12 s) or the bursting phases (mean: 2 s), respectively, did not vary with circadian time. Similarly, the proportion of cells belonging to a specific cell group remained unchanged during the whole duration of the experiment.

In the SCN, it is known that the effect of a resetting stimulus which occurs at the time of slice preparation is preserved in the SCN *in vitro* [18]. Therefore, we additionally plotted the data depending on the time at which the animals were sacrificed for slice preparation and also under these conditions we did not observe any difference in the average firing rate over the course of a 24 hours period (p>0.3; Fig. 2B).

### Whole-cell Recording of Post-synaptic Activity

**Figure 3:**
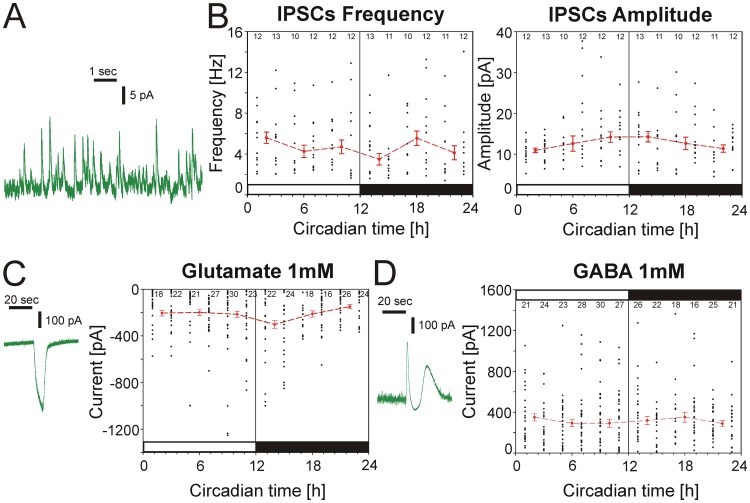
No circadian modulation of the synaptic inputs to Purkinje cells in acute brain slices. **A)** Whole-cell patch-clamp recording from a Purkinje cell showing inhibitory post-synaptic currents (IPSCs) under a holding potential of −50 mV. **B)** The median of frequency (left) and amplitude (right) of the IPSCs did not vary over a 24 hours cycle (p>0.05). Single black dots represent the average amplitude or frequency of IPSCs for every single Purkinje cell recorded for 5 minutes. Red squares with error bars with the broken line represent the median of the frequency or amplitude of IPSCs from all Purkinje cells recorded during a four hours period. **C)** The amplitude of glutamate-induced currents recorded in four hour bins did not vary over a 24 hours period (right) (p>0.05). Left: recording trace of a glutamate-evoked current in voltage-clamp. **D)** The amplitude of GABA-evoked currents showed no change with circadian time (right) (p>0.05). Left: recording trace of a GABA-evoked current in voltage-clamp.

To examine a possible circadian modulation of the excitatory and inhibitory input pathways into the Purkinje cells we determined the synaptic currents on cerebellar Purkinje cells. *In situ*, Purkinje cells receive excitatory inputs from glutamatergic granule cells and olivary neurons via parallel and climbing fibers, and inhibitory inputs from basket and stellate cells, but in slice culture the two excitatory afferents are absent [28]. In order to examine a possible circadian modulation of synaptic activity we determined changes in spontaneous synaptic events in Purkinje cells over the course of the day. We recorded inhibitory postsynaptic currents (IPSCs) in whole-cell configuration at a holding potential of −50 mV. Spontaneous activity was recorded in a single cell for several minutes before the next cell was examined. However, neither the frequency (p>0.1; 5.10±0.26 Hz; n = 13) nor the amplitude (p>0.4; 13.45±0.53 Hz; n = 13) of IPSCs significantly varied with time of the day (Figure 3A and B) and also the amplitude of GABA- (p>0.9; −198.14±9.79 pA; n = 30) and glutamate- (p>0.1; 349.24±15.58 pA; n = 30) evoked currents were stable over the course of the day when both neurotransmitters were applied exogenously (Figure 3 C and D).

### Multielectrode Recordings

The variability in firing activity between individual animals and the absence of a rhythm in random single cell recordings from acute slices prompted us to perform recordings in organotypic cultures with the help of MEAs. Organotypic cultures preserve the structural and physiological characteristics described *in vivo* and are together with the MEA technique an excellent method to monitor extracellular circadian activity on many electrodes simultaneously for prolonged periods lasting days or weeks [19], [20], whereas single electrode recordings from acute slices are limited to minutes or hours. If only a small subset of Purkinje cells or other cerebellar cells contains a self-sustained oscillator, the rhythm cannot be detected in random measurements of the average firing rate by single electrodes, but will be feasible by continuous recordings from individual cells on MEA.

**Figure 4:**
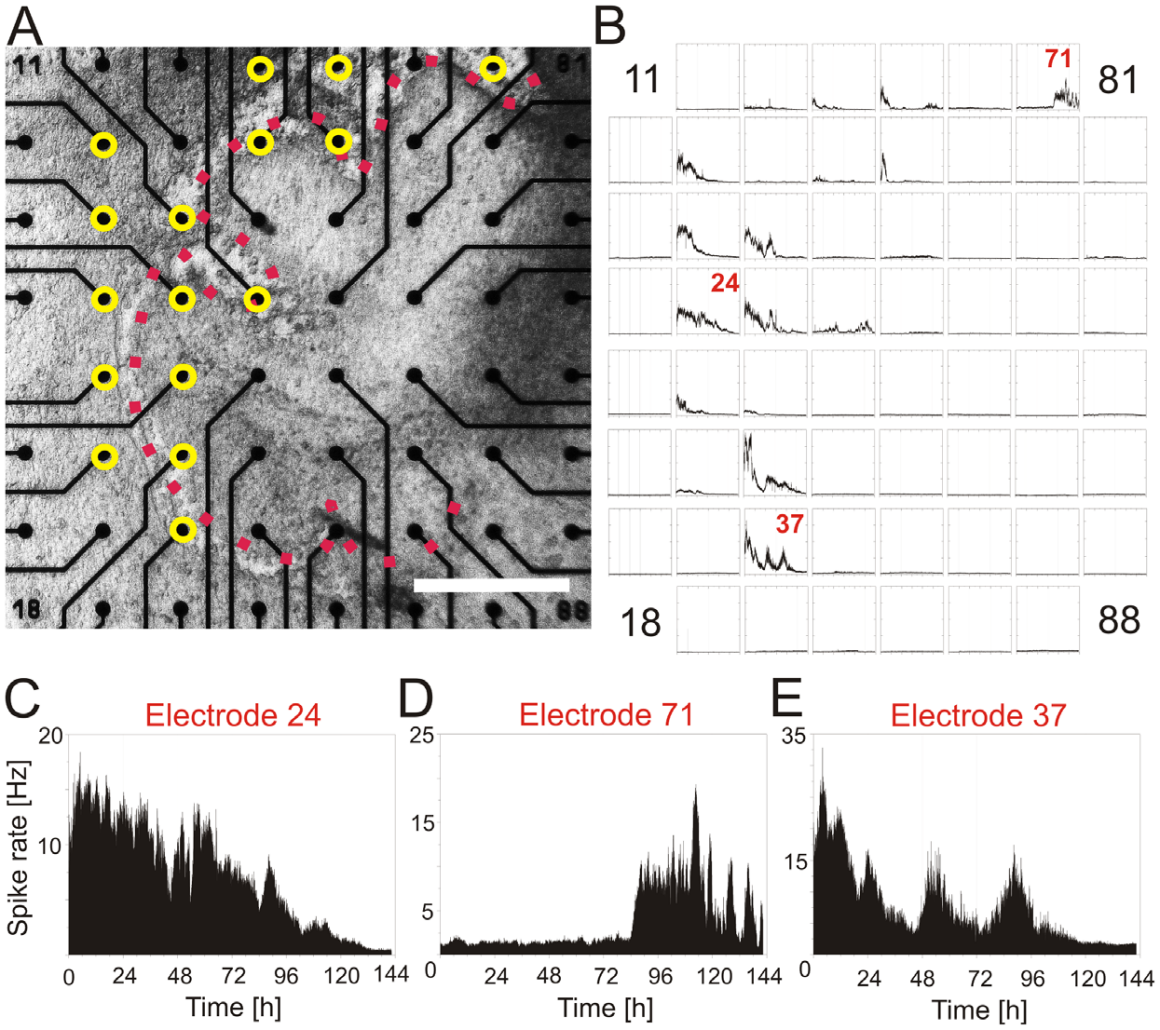
MEA recordings from an organotypic cerebellar slice. **A)** Photomicrograph of an organotypic sagittal cerebellar slice on an 8×8 MEA. The Purkinje cell layer is indicated by the red squares. Yellow circles superimposed on the figure indicate those electrodes that show a spontaneous activity and spike pattern similar to Purkinje cells. All of these electrodes are situated close to the Purkinje cell layer. Scale bar: 400 µm. **B)** Recording of spike rate for 144 hours for the 60 electrodes represented in (**A**). At most electrode positions, no clear circadian oscillation of the Purkinje cell firing rate could be observed for several cycles during the recording period of 6 days. However, some electrodes (eg. electrode 33, 34) showed for one cycle a 24 hour oscillation which was not maintained for longer periods. Electrodes that were not covered by the Purkinje cell layer showed only a very low spike activity. The scale on the ordinates is set to a maximum of 40 Hz for each channel. **C)**, **D)** and **E)** Magnification of the three marked graphs in (**B**) showing typical examples of the firing rate of individual Purkinje cells. **C)** In the majority of recordings, the initial high spike rate declined progressively after a few hours (electrode 24) until it disappeared at the sixth day. **D)** In other cases, a previously silent cell started firing after several days of recording (electrode 71). **E)** Oscillations of the firing rate were evident in a low number of recordings from Purkinje cells before they damped out or disappeared after few cycles. The period of the three-day long oscillations was 32.6 hours (electrode 37). The numbers at each corner of the electrode field (**A**) and the graphs (**B**) indicate the numbering in the MEA layout.

We performed MEA recordings from sagittal slices kept 2–5 weeks in culture. Figure 4A shows recordings from many regions of the cerebellar cortex. The Purkinje cell layer is clearly visible in this slice and marked by red squares. Yellow circles superimposed on the figure represent the electrodes with a clear spontaneous activity of high amplitude as it is indicative for Purkinje cells. Generally, the firing rate of Purkinje neurons was in the range between 5 and 50 Hz, with an average rate of 12.5±2.2 Hz (min: 1.1 Hz, max: 77 Hz), similar to the only other study performing long-term MEA recordings from Purkinje cells [23]. The typical firing pattern with tonic (Fig. 1C) and trimodal-like pattern (Fig. 1D), as observed in single cell recordings from acute slices (Fig. 1A and B), are a further characteristic indicating that these spikes originate from Purkinje cells. After discrimination of single cells by spike sorting, single neurons displayed a highly autocorrelated activity showing a regular firing pattern with multiple clearly defined peaks. A comparison of autocorrelograms obtained for the two main firing pattern in MEA and in single electrode extracellular recordings shows a similar shape of the spike trains, only the number of oscillations being larger and more precise in acute slices compared to organotypic slices, and the time interval between two oscillations was shorter in acute slices (75 ms vs. 110 ms; Fig. 1). Immunostaining with an antibody against calbindin after the end of the experiments provided further evidence that the spikes analyzed in MEA recordings originate from Purkinje cells (see Fig. S1). In some cases, cells located close to the Purkinje cell layer displayed bursting activity or random firing patterns; these cells could not be identified with certainty as Purkinje cells.

### Changes of the Firing Rate during a Circadian Cycle

In long-term MEA recordings, spike activity showed high variability, usually with high firing rate at the beginning of the experiments, which was not maintained for long periods. In these cases, spike activity decreased or completely disappeared after 2–10 hours; other cells were silent at the beginning of the experiments, but started firing after a few hours or sometimes days (for example electrode 71 in Fig. 4D).

However, the time course of the firing rate showed in some cases oscillations with periods between 21.3 hours and 33 hours for 3 to 5 cycles (see electrode 37 in Fig. 4). The average period of these rhythmic cells was 24.27±0.65 hours (n = 17, out of more than 300 recorded cells). All of these cells appear to belong to the Purkinje cell population as judged from their position in the slice and their firing pattern. Nevertheless, the periods varied from day to day and the cyclic activity disappeared after few days.

### Medium Replacement can Induce a Circadian Rhythm

**Figure 5:**
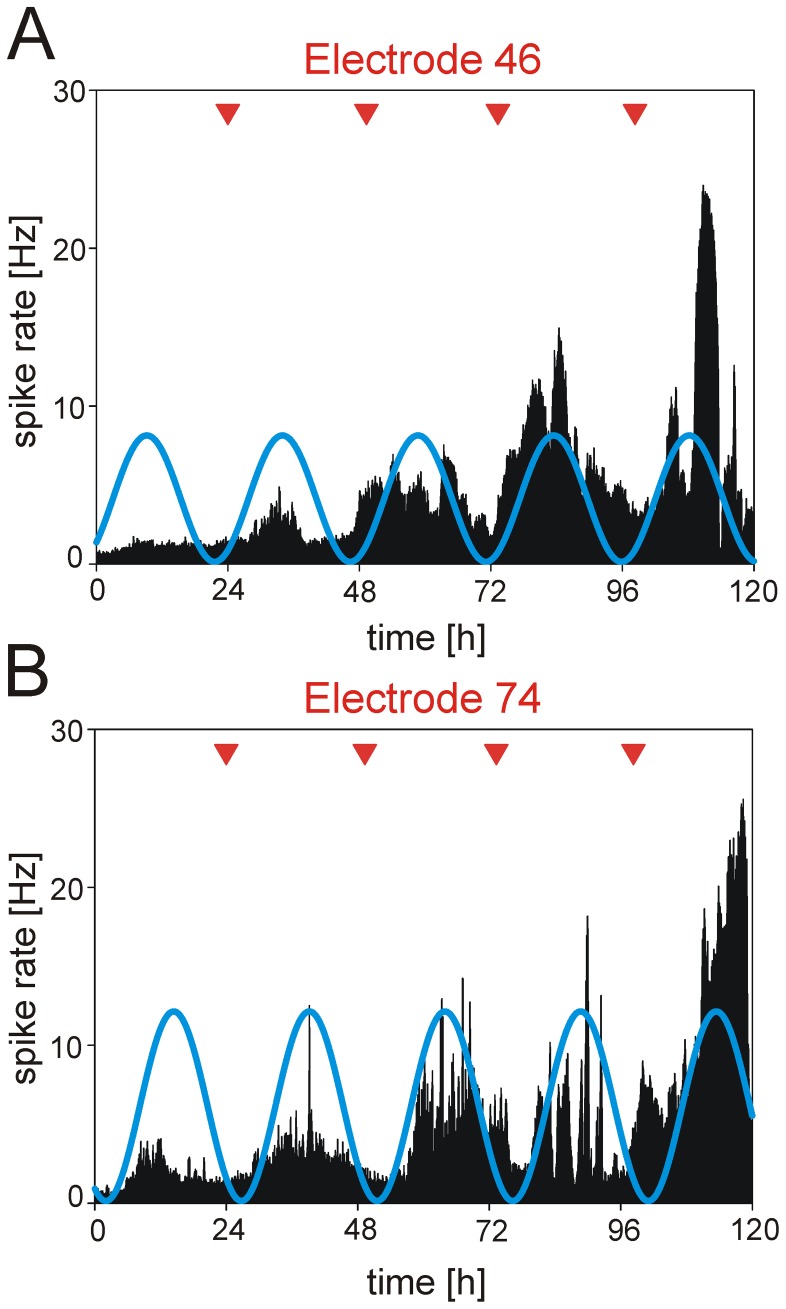
Medium replacement can induce a circadian rhythm in Purkinje cell firing rate. **A)** and **B)** represent extracellular recordings of the spike rate from two different cells (electrode 46 and 74) obtained from a slice cultured on a standard MEA. Red triangles show the time of renewal of the medium in daily intervals (between 24 and 25 hours). The exchange of the medium with fresh medium induces a rhythm in the neuronal activity of the Purkinje cells that closely followed the timed renewal. However, the rhythm induction was not accompanied by a synchronization of the spike rate because the rhythm in both cells was out of phase. The blue curve depicts a cosine curve fitted to the recording traces.

Peripheral clocks require, contrary to the master pacemaker in the SCN, external signals to sustain or synchronize their internal circadian rhythms. In order to investigate whether medium replacement can induce a circadian rhythmicity in Purkinje cells, we exchanged the recording medium in MEA recordings in regular intervals close to 24 hours against freshly prepared medium. In all other long-term experiments, medium was randomly exchanged in time intervals of about 3 days to ensure relatively undisturbed recording conditions. However, when the medium was replaced in intervals of 24–25 hours, rhythmicity in spike activity was induced in several Purkinje cells for up to five days. Figures 5A and B show such a MEA experiment with a medium renewal every 25 hours showing a clear and significant induction of a rhythmicity of spike discharges for five days. The phases calculated for both cells were 24.8 hours (Fig. 5A) and 25.0 hours (Fig. 5B), respectively, i.e. they closely coincide with the renewal interval. However, the two cells shown in Fig. 5 clearly exhibit a rhythm induction (Fig. 5A) or a rhythm amplification (Fig. 5B), but, interestingly, the rhythms are 5.6 hours out of phase suggesting that medium renewal leads to an induction of rhythmicity, but not to a synchronization, and that the rhythm was not induced by a mechanical disturbance of the neuron which should induce similar variations in the activity of all other neurons.

## Discussion

It is a fundamental question whether rhythmic clock gene expression in the brain or in peripheral tissues is, similarly to the master pacemaker in the SCN, transduced into an electrical output to communicate the circadian signal to its appropriate target. One of the brain areas showing oscillations in clock gene expression is the cerebellum [13] which was, until recently, not regarded as a major component of the circadian timing system. However, the question is still open whether cerebellar circadian clock gene expression is transduced into an appropriate rhythmic output signal. Using the established method to detect circadian rhythmicity in an *in vitro* SCN slice preparation by randomly recording spike activity over a time period of 24 hours [26], we monitored the electrical output signals from acute cerebellar slices, but did not find clear evidence for a circadian variation of the mean firing rate of acute slices. All these recordings in acute slice preparations were unambiguously performed from Purkinje cells, which form the sole output of the cerebellar cortex [27], as judged by visual identification of the Purkinje cell layer above the electrodes, the typical spike pattern and the specific staining following recordings. As we did not find any circadian modulation of spontaneous inhibitory postsynaptic currents, the endogenous cerebellar pacemaker also does not shape the synaptic input into Purkinje cells, as it is observed in SCN neurons in GABAergic neurotransmission [29]. Although we recorded from a relatively large number of Purkinje cells over the time course of the 24 hour cycle, this method gives only information about the mean activity of a randomly selected cell population, but no information about the behavior of individual cells. In the SCN with its numerous clock cells, the method gives a reliable measure of SCN population activity and the clock output *in vitro* [18], [26] and is congruent with data of rat SCN multi-unit activity obtained *in vivo* [15], but the method is apparently not suitable to detect clock driven oscillations in the cerebellum. This drawback was overcome by the use of MEA, which allow long-term recordings from multiple single cells with a time span that was previously not applicable in electrophysiological recordings. Even in these MEA recordings, the majority of spontaneously spiking cells did not exhibit clear circadian oscillations of their firing rate, showing that the principal output of the cerebellum is not under the control of a circadian oscillator. However, multisite MEA recordings revealed several spontaneously active cells, presumably Purkinje cells, with a rhythmic modulation of their spike rate with periods of about 24 hours or slightly longer, but the rhythms damped rapidly after one or two cycles. Interestingly, rhythmicity could be induced in some previously non-rhythmic Purkinje cells by a regular external stimulation, a feature that is known from slave oscillators where circadian oscillations in gene transcription are not sustained, but can be reinstated with regular, cyclic stimulation [30]. Unlike self-sustained oscillators, the rhythm of slave oscillators dampens and disappears after several days *in vitro* [2]. This was also observed in the cerebellum of rats and mice where bioluminescence measurements of Per1- and Per2-luciferase show a constant dampening of the bioluminescence intensity with time [3], [13]. This suggests that the cerebellum functions more as a slave oscillator and requires an input from a master clock to maintain strong circadian rhythmicity. However, this poses the question about the nature of rhythmic action potential firing. In SCN neurons the ionic mechanisms underlying action potential firing rhythms, although not completely understood, appear to involve an array of different ionic channels which are under the control of the circadian clock and promote high daytime firing (for review see [31]). In Purkinje cells of the cerebellum, spontaneous activity also results from intrinsic conductance and seems to be largely independent of synaptic transmission, but it is possible that the ionic conductance that is responsible for generation of spontaneous activity might, contrarily to the SCN, not be under the control of the molecular clock.

Purkinje cells are the sole output of the cerebellar cortex circuitry and provide the signals that are required for planning, execution and coordination of motoric functions [12]. They are additionally involved in short temporal processing as a kind of interval timer in the range of tens to hundreds of ms [32]. The rhythmic clock gene expression in the cerebellar cortex suggests an involvement of the cerebellum in longer temporal processing, i.e. in circadian processes [3]. It was shown that this circadian timer in the cerebellum is sensitive to feeding cues, because during meal anticipation, glucose utilization is reduced in the cerebellum [33], and clock gene oscillations are shifted in response to restricted feeding [13]. Food-anticipatory activity was significantly reduced in the study of Mendoza et al. [13] by destroying the Purkinje cell layer with an immunotoxin, and also in mutant mice with impaired cerebellar circuitry, indicating that the cerebellum, and especially Purkinje cells, is required for the anticipation of mealtime. However, if a circadian pacemaker resides in Purkinje cells or in any other cerebellar cell structure, the question remains how this circadian information is transduced into a signal that is relevant for the food-entrainable clock and its related food anticipatory mechanisms and that is transmitted to the appropriate brain targets. The anatomical substrate of the FEO is composed of a network of interconnected brain structures rather than a single brain area driving food entrained circadian rhythms [34]. There is some evidence that it is located outside of the SCN because food anticipatory behavior is still present in SCN-lesioned animals [35]. Many regions in the thalamus, hypothalamus, and forebrain exhibit alterations in clock gene expression under temporary food restriction, including the paraventricular thalamic nucleus, the dorsomedial nucleus of the hypothalamus, hippocampus, lateral septum, nucleus accumbens, and cerebral cortex [34]. As part of this network, Purkinje cells need to communicate with at least a part of these structures with specific signals that could be similar to those described in the master clock in the SCN. In the SCN, circadian information is either transduced into an electrical or a humoral output which can influence the targets of the circadian system in the brain or in the periphery (for review, see [14]). Humoral factors alone are sufficient to restore circadian rhythms in locomotor activity in SCN-lesioned animals by implanting fetal SCN [36], whereas circadian neuroendocrine rhythms appear to require intact neural projections [37].

Humoral factors are also essential to regulate food ingestion, but there is presently, at least to our knowledge, no evidence that the cerebellum releases any hormone or humoral signal in a circadian fashion. Several humoral factors are involved in food entrainment synchronizing feeding behavior with a specific time of the day. These factors include the orexigenic ghrelin which is released from the stomach and acts on ghrelin receptors in the arcuate nucleus and other parts of the hypothalamus. Ghrelin is related to, but not essential for anticipation of food availability [38]. Another hormone related to food ingestion is leptin released by adipose tissue which may inhibit food anticipation [39]. Leptin receptors are expressed in high density in the cerebellum and their expression is downregulated by high-fat feeding suggesting that peripheral leptin transmits a metabolic signal to the cerebellum [13]. The leptin signal converges also on neurosecretory TRH neurons of the hypothalamic paraventricular nucleus together with alpha-melanocyte-stimulating hormone and neuropeptide Y signaling. Since TRH-like neuropeptides show in the cerebellum diurnal rhythms of their concentration, this could be another indirect pathway for the transmission of diurnal signals to the cerebellar cortex [40]. Other humoral signals involve the glucose-insulin-glucagon pathway. Under restricted feeding schedule, rats show an increase of glucagon and a decrease in insulin before food access [41]. Since glucose transporters (Glut) including the insulin-responsive Glut4, are expressed in the cerebellum [42], this might be also a pathway by which feeding cues can influence cerebellar functions. All these humoral signals that are involved in the transmission of feeding cues and the FEO network can provide an afferent input to the cerebellum, but none of them is known to provide an output signal of the cerebellar clock mechanism that could influence the FEO.

If the cerebellar clock transmits its signals via a rhythmic electrical activity in Purkinje fibers, the question remains why only few cerebellar neurons exhibit signs of a circadian neuronal activity? All investigations in the cerebellum were performed *ex-vivo*. It is known from investigations from the SCN that synchronization between neurons depends strongly on the network organization increasing the precision of the clock [30], [43]. In the cerebellum, a neuronal network exists in organotypic cultures similarly to acute slices, even in the absence of the excitatory afferent input by mossy fibers and climbing fibers [28]. However, from calbindin immunocytochemistry, a specific marker for Purkinje cells, it appears that in organotypic slices a considerable number of cells die in the first week of culture. Staining with calbindin after the termination of MEA recordings show gaps in the Purkinje cell layer, and, in some cases, single Purkinje neurons were disseminated in the slice and their axons seemed to extend to random directions (data not shown). This might indicate a partial loss of this cell type during culture and consequently an altered neuronal network which could explain the low number of rhythmic firing cells which could belong to a more fragile Purkinje cell subtype. Although the neuronal activity in cultured cerebellum retains their physiological features, slice explants possess a number of characteristic architectural properties. A proportion of Purkinje cells lose the usual polarity observed *in vivo* in the cerebellar cortex, and in the absence of the deep cerebellar nuclei within the culture, Purkinje fibers return toward their cell layer, innervate the molecular layer or are randomly distributed [28]. All these changes could lead to a loss of synchronization in the cerebellum which is necessary to express and maintain a circadian rhythmicity.

The cerebellum is organized in different compartments with their own phenotypic characterisation. Aldolase C (or zebrin II) has been described to form bands of expression in the different lobules of the cerebellum [44]. This compartmentation could reflect functional localized specializations in the cerebellar cortex as axon collaterals from Purkinje cells are forming synapses only with cells belonging to the same zebrin-positive region of the cerebellar cortex [45]. It appears that cerebellar cortical excitability, information processing, and synaptic plasticity depend on the intrinsic properties of different parasagittal zones in the cerebellum [46]. Moreover, the deep cerebellar nuclei integrate the output signals from Purkinje cells following this compartmentation [47]. Their readout is based on sparse coding and population coding. A circadian signal might then be coded by a specific ensemble of Purkinje cells rather than multiple individual neurons. If only a subset of these compartments express circadian oscillations, random recordings from a slice cannot uncover rhythmicity. The MEA technology with its simultaneous multisite recordings for long periods detected only in few cases oscillations of about one day. A recording field with 60 electrodes is more effective to discover a special cell type compared to single electrode recordings, but apparently not sufficient to provide information about the behaviour of the complete Purkinje cell layer with all its zoning. However, MEA recordings are a clear improvement in respect to the number of simultaneous recordings and long-term stability of such recordings.

A second facet concerns a possible altered maturation of the cerebellar network in organotypic slice cultures compared to *in vivo* conditions which might impair circadian cerebellar function. Organotypic slices are usually prepared from newborn mice, P0 to P2, because in older animals the survival rate of the slices is considerably impaired [48]. The mouse cerebellar cortex requires for maturation about 14 to 21 days after birth. Therefore it is possible that some developmental factors that are essential for a correct formation of the network are missing. During development, granule cells migrate in the first two postnatal weeks from the external granular cell layer to the internal granule cell layer [49]. This migration is possibly impaired in organotypic slices because we observed in four week old cultures granular cells also near the molecular layer and not only in the internal granule cell layer (data not shown) which could speak for an insufficient maturation of the cerebellar network. In this context, it is noteworthy that the pineal hormone melatonin, as a component of the circadian timing system, possesses many binding sites in the cerebellum [50] and constitutes a neurotrophic factor that is essential for controlling cerebellar granule cell fate [51].

Cerebellar granule cells themself show clock gene expression, but *Per1* expression requires several signaling pathways like Ca^2+^ influx or activation by PACAP [52], and thus, they appear to be more slave oscillators rather than master oscillators. Granule cells receive excitatory input from mossy fibers originating from pontine nuclei, which themselves display an expression of clock genes [53] and could also provide a rhythmic signal to the cerebellar cortex. Other parts of the brainstem, such as the nucleus of the solitary tract, expressing clock genes, also project to the cerebellum and are likely associated with the FEO [9]. However, cerebellar modulation of visceral functions does not only rely on afferent and efferent connections with the brainstem, but involve also bidirectional connections between the cerebellum and the hypothalamus [54]. The neurotransmitters in the hypothalamo-cerebellar pathway, although not well known so far, involve histamine, GABA, and glycine [55]. Serotonin, synthesized in brainstem, is another modulator of Purkinje cell activity linked to regulation of feeding and arousal. These transmitter candidates are all implicated in circadian functions and show either circadian or diurnal rhythms of their brain level and/or act directly on the master clock in the SCN by phase shifting the rhythms [20], [30], [56].

In conclusion, despite rhythmic clock gene expression in the cerebellum and the recent implication of the cerebellum in a food entrainable network, a clear circadian modulation was not detected for the spike rate or synaptic transmission of the majority of Purkinje cells *in vitro.* However, a fraction of cerebellar output neurons show characteristics of slave oscillators; their rhythm damps after few days and rhythmicity can be induced by external stimulation. This is supported by a recent study showing that rhythmic clock gene expression in the cerebellum is lost in rats with SCN lesions. Since direct neuronal projections linking the SCN to the cerebellum have not been described, it was assumed that this influence could be due to secondary changes in circadian physiology [57]. The intimate, bidirectional relationship between cerebellum, hypothalamus and circadian system for the control of food-anticipatory activity must be further elucidated to identify the feeding-associated circadian network.

## Supporting Information

Figure S1 **A)** Organotypic cerebellar slice on a high-density MEA field. The figure shows an immunostaining for calbindin D28K to label somata, dendrites and axons of Purkinje cells (green). Scale bar 60 µm. **B)** Scheme of one field of 30 electrodes with their identification by letters (lines) and numbers (columns). **C)** Example of a recording obtained from the organotypic slice shown in **(A)** on electrode E1. Top: superimposed spikes of a single cell after spike discrimination (left) and the corresponding spike train over a period of about 30 s (right). Bottom: autocorrelogram drawn for all spikes recorded during a period of 4 hours (left). The spike rate was stable over longer time periods (right).
(TIF)
