# Supplementary figures and images for: The Output Signal of Purkinje Cells of the Cerebellum and Circadian Rhythmicity
Source: PLoS One. 2013 Mar 7;8(3):e58457. doi: 10.1371/journal.pone.0058457 (PMC3591352; doi:10.1371/journal.pone.0058457)

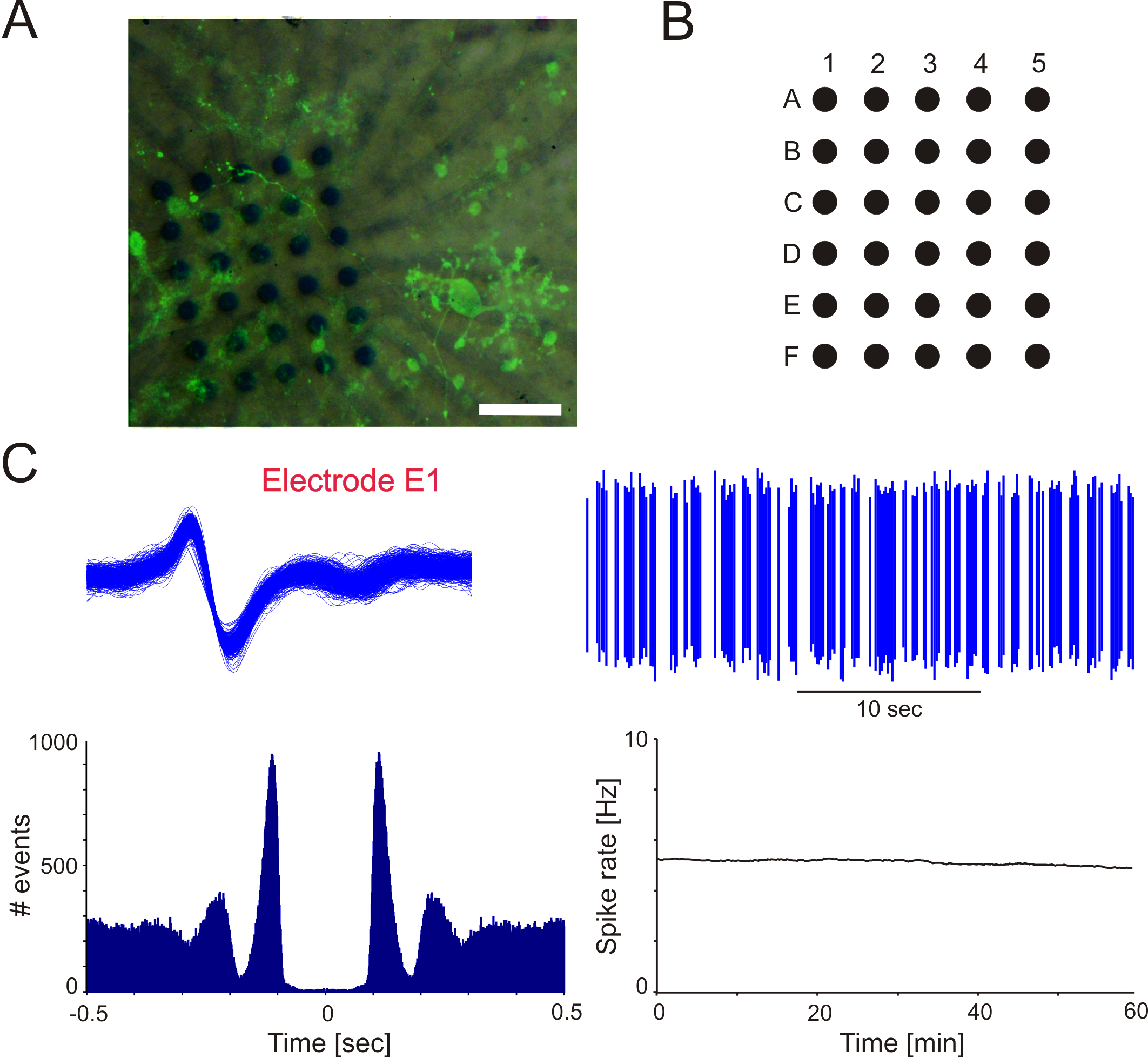

Supplement: Figure S1 — A) Organotypic cerebellar slice on a high-density MEA field. The figure shows an immunostaining for calbindin D28K to label somata, dendrites and axons of Purkinje cells (green). Scale bar 60 µm. B) Scheme of one field of 30 electrodes with their identification by letters (lines) and numbers (columns). C) Example of a recording obtained from the organotypic slice shown in (A) on electrode E1. Top: superimposed spikes of a single cell after spike discrimination (left) and the corresponding spike train over a period of about 30 s (right). Bottom: autocorrelogram drawn for all spikes recorded during a period of 4 hours (left). The spike rate was stable over longer time periods (right). (TIF) [file pone.0058457.s001.tif]
